# Comparison of impact on death and critical care admission of acute kidney injury between common medical and surgical diagnoses

**DOI:** 10.1371/journal.pone.0215105

**Published:** 2019-04-11

**Authors:** Lynne Sykes, Philip A. Kalra, Darren Green

**Affiliations:** 1 Emergency Assessment Unit, Salford Royal NHS Foundation Trust, Stott Lane, Salford, United Kingdom; 2 Renal department, Salford Royal NHS Foundation Trust, Stott Lane, Salford, United Kingdom; University of Mississippi Medical Center, UNITED STATES

## Abstract

**Background:**

Acute Kidney Injury (AKI) is common and associated with increased morbidity and mortality. This retrospective analysis quantified and compared the association between AKI and the risk of death and admission to critical care in acute admissions of different aetiology.

**Methods:**

Data were extracted anonymously from the Trust ‘data warehouse’ for admissions between 2011and 2017. We applied KDIGO AKI criteria to establish AKI stage. Odds ratios (OR) for death and critical care admission were calculated for patients with AKI stage 3 (compared to all other patients), and patients with any stage AKI (compared to non-AKI admissions). Analyses were performed using logistic regression, adjusted for age, pre-existing CKD, co-morbid index, and gender.

**Results:**

There were 26,052 medical and 12,560 surgical patient episodes within sixteen common diagnoses with 3823 medical and 1520 surgical patients with AKI events. The likelihood of AKI was highest in sepsis (31.8%), and the likelihood of death in AKI 3 highest in femoral neck fracture (54.5%). AKI 3 has a OR for death for acute coronary syndrome of 12.8 and a OR of 24.6 in femoral neck fracture. Admission to critical care for any AKI in medical patients has a OR of 9.6, but increases to OR 37.2 for heart failure.

**Conclusion:**

The clinical impact of AKI differs across medical and surgical diagnoses, but is a significant contributor to the risk for death and critical care admission. This body of work may indicate a benefit to a more diagnosis-specific stratified approach to AKI care.

## Background

Acute Kidney Injury (AKI) is a common and serious condition that is associated with increased morbidity and mortality.[1–3] It is not a disease but rather a syndrome and a reflection of the severity of an illness affecting a patient.[4] Increasingly therefore, AKI can be used as an ‘illness barometer’ for patients. An episode of AKI has strong associations with increased length of stay, mortality, level of care, and specialty input required over the course of admission.[5–7]

AKI affects a wide range of patients both within hospital and in primary care. The incidence of AKI in hospital under different specialty teams (excepting nephrology) is highest in medicine for the elderly, cardiology, general surgery and gastroenterology. Within the NHS AKI cases are managed by the specialty team managing the main medical or surgical condition rather than specifically by nephrologists[8], although the latter do supervise the care of patients with the more severe AKI episodes.

Previous studies into AKI in different specialties have used the admitting specialty as an umbrella proxy to categorize patients into groups, and as such may lack the granularity to understand patient complexity and confounders such as specific diagnoses and co-morbidities.[9,10] This latter consideration may be vital because diagnosis is likely to be more accurate than the umbrella parent specialty in stratifying individual patient risk associated with an AKI episode.

The aim of this study was to quantify and compare AKI epidemiology in secondary care between specific common medical and surgical diagnoses. The intent was to evaluate not only the incidence of AKI, but also the impact that AKI has on outcomes after hospital admission, specifically death and critical care admission. Such information may allow adoption of a more patient-specific, stratified approach to AKI care by recognizing that the prognosis after AKI will differ between diagnoses.

## Methods

### Setting

Salford Royal NHS Foundation Trust is a large teaching hospital in the North West of England. It has 820 inpatient beds and covers a direct catchment population of 220,000. It is a Major Trauma Centre, provides tertiary stroke, dermatology, and neurosciences care for the Greater Manchester area (population 3.5 million), and is a tertiary referral centre for renal medicine for approximately 50% of this conurbation. It is a national centre for patients with intestinal failure and metabolic diseases. Neighbouring NHS Trusts provide tertiary services for cardiothoracic surgery, vascular surgery, and cardiac catheterization, including for patients from the Salford catchment area.

### Data

Data for all non-elective inpatient episodes at Salford Royal between 1^st^ March 2011 and 31^st^ December 2017 were extracted anonymously from the Trust ‘data warehouse’. Salford Royal is a global digital exemplar site and data extraction relating to patient episodes could be performed with complete patient anonymization and with a high level of granularity for event data. Data extraction was performed as part of an AKI quality improvement initiative. This was exempt from specific ethical approval as it was anonymised data. [11]

Data extracted were date of admission, length of stay, critical care admission, age, gender, ICD-10 codes for admission diagnosis and co-morbidities, inpatient mortality, dialysis episodes, and laboratory data for serum creatinine values. The 8 most common acute medical and surgical admission diagnoses were selected based on ICD-10 codes determined after discharge, and are shown in Table 1. In order to acknowledge that some patients have more than one diagnosis during admission, patients were grouped according to their coded main diagnosis. Patients admitted with other diagnoses were excluded, as were patients already established on maintenance dialysis therapy or those who had a functioning renal transplant.

**Table 1:** The 8 most common medical and surgical diagnosis categories and their relative frequency amongst all admissions.

Medical			Surgical		
Key	Diagnosis	Frequency %	Key	Diagnosis	Frequency %
ACS	Acute coronary syndrome	2.1	Abscess	Abscess (any site)	0.7
ALD	Alcoholic liver disease	0.3	Appendix	Acute appendicitis	0.3
CAP	Community acquired pneumonia	3.6	Chole	Cholecystitis/ cholangitis	1.1
COPD	Chronic obstructive pulmonary disease	2.1	ENT	ENT (ears, nose and throat) (any source)	0.3
GIB	Gastro-intestinal bleed	0.8	NOF	Femoral neck fracture (NOF)	0.9
HF	Heart failure	0.8	NTICB	Non Traumatic Intra Cranial Bleed	1.4
Sepsis	Sepsis (any source)	0.8	Panc	Acute pancreatitis	0.5
UTI	Urinary tract infection	2.4	TICB	Traumatic Intra Cranial Bleed	1.0

The central pathology laboratory of the Trust provides biochemistry services for all inpatient and outpatient venous samples, including those taken in a primary care setting. From March 2011 until January 2015, a compensated kinetic Jaffe method with an inter-assay coefficient of variance of 1.7% at 193 umol/L (Roche Cobas 8000) was used to measure all serum creatinine values (normal creatinine range, male: 62–106 μmol/L; female: 44–80 μmol/L). From January 2015 to December 2017 the method was a compensated kinetic Jaffe method with an inter-assay coefficient of variance of 2.9% at 156 μmol/L (Siemens, Advia) (normal creatinine range, male: 62–115 μmol/L; female: 44–97 μmol/L). The Kidney Disease Improving Global Outcomes (KDIGO) AKI criteria[12] were manually applied retrospectively using the National AKI algorithm[13] to available creatinine measurements to determine AKI episodes during each admission, and to establish AKI stage.

### Statistical analysis

A comparison of patient characteristics was made between medical patients with diagnoses selected for inclusion in the study, surgical patients with diagnoses selected for inclusion in the study, and the remainder of admissions during the study period, except for those fitting exclusion criteria as detailed above. Inter-group comparison of binary variables was performed using chi-square tests and comparison of continuous variables was performed using one-way ANOVA.

For each selected admission diagnosis, Odds ratios (OR) were calculated for inpatient death and for admission to critical care after AKI onset in patients with AKI stage 3 compared to other patients, including those with lesser stages of AKI. The analyses were repeated comparing outcomes for patients with any stage of AKI in comparison to admissions without AKI for that specific diagnosis.

All of the analyses were performed using logistic regression, adjusted for age, pre-existing chronic kidney disease (CKD), co-morbid index, and gender. IBM Statistical Package for the Social Sciences (SPSS) version 23 for Mac [SPSS (UK) Ltd, Woking, Surrey, UK] was used for analyses.

## Results

### Study population

Between March 2011 and December 2017 (80 months) there were 197,884 non-elective inpatient episodes. Of these, 26,052 (13.2%) fell within the 8 selected medical diagnoses, and 12,560 (6.3%) within the 8 selected surgical diagnoses. The most common medical diagnosis was community-acquired pneumonia (n = 7,323), which accounted for 3.6% of all hospital admissions during the study period. The most common surgical diagnosis was non-traumatic intracranial bleed (NTICB, n = 2,831), which accounted for 1.4% of admissions. Overall, the selected medical and surgical diagnoses accounted for 19.1% of all hospital admissions during the study period. Full details of the selected diagnoses and their frequencies are found in Table 1.

The split between male and female patients in the medical and surgical selected diagnoses (both 52% female) was more even than in the rest of the acute admission population (57% female). The selected medical population had a higher mean age, number of co-morbidities, CKD incidence, AKI and AKI 3 incidence, mortality, and critical care admission than both the overall acute admission population and selected surgical population. The selected surgical population was older, had a higher number of comorbidities, AKI incidence and death rate than the overall acute admission population, but lower AKI 3 incidence. Full details of comparisons between the selected and whole populations are found in Table 2.

**Table 2:** Demographics of the selected medical and surgical diagnoses in comparison to the overall admission population.

Demographic	Overall	Medical selected diagnoses	Surgical selected diagnoses
Patients	197,884	26,052 (13.2%)	12,560 (6.3%)
Male gender (%)	85,090 (43%)	12,558 (48%)	6076 (48%)
Age mean (SD)	55.5 (22.3)	69.8 (16.9)	57.3 (21.7)
CKD (%)	17,262 (8.7%)	3283 (12.6%)	545 (4.3%)
Comorbidities (range)	6.9 (1–17)	9.6 (1–17)	8.3 (2–17)
Any AKI (%)	15,217 (7.7%)	3823 (14.7%)	1520 (12.1%)
AKI 3 (%)	5740 (2.9%)	638 (2.4%)	85 (0.7%)
Death (%)	6749 (3.4%)	2292 (8.8%)	974 (7.8%)
Critical care (%)	9001 (4.5)%	606 (2.3%)	1852 (14.0%)

### Event counts for acute kidney injury by medical and surgical diagnosis

**Fig 1:**
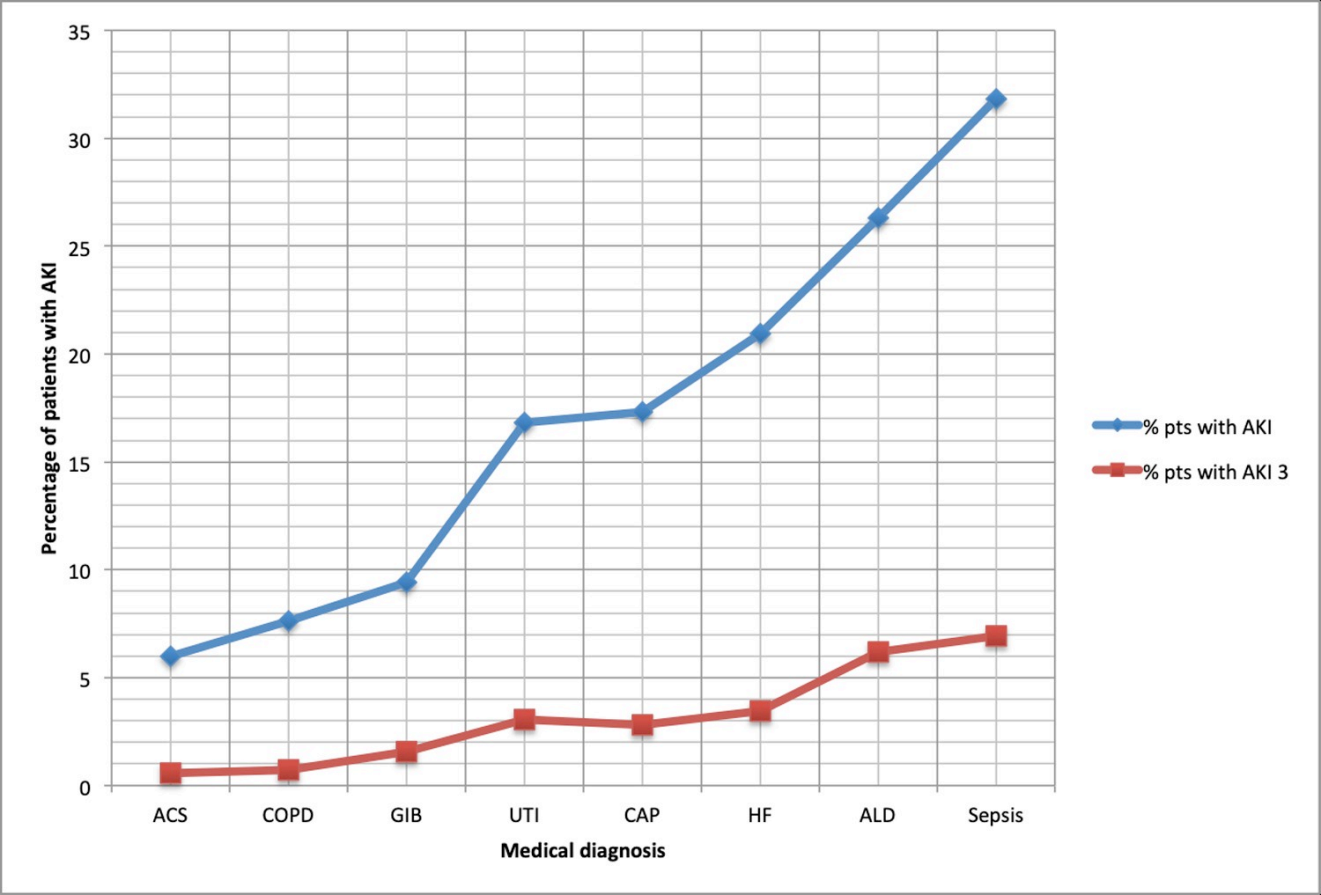
Percentage of patients with any acute kidney injury (AKI) or AKI 3 in each medical diagnosis, ordered by increasing frequency of AKI. **Figure key:** HF–heart failure, ACS–acute coronary syndrome, CAP–community acquired pneumonia, COPD–chronic obstructive pulmonary disease, UTI–urinary tract infection, ALD–alcoholic liver disease, GIB–gastro-intestinal bleed, sepsis–sepsis (any source). % pts with AKI = percentage of patients with each diagnosis with AKI or AKI 3.

In the whole acute admission population there were 197,884 patient episodes with 15,218 AKI events (7.7%) as shown in Table 3. There were 26,052 patient episodes within the 8 selected medical diagnoses, with 3,823 (25.1%) AKI events. There were 638 (4.2% of the whole pop, 16.7% of the medical AKIs) AKI stage 3 events. The likelihood of any AKI was highest in sepsis (31.8%), and lowest in acute coronary syndrome (ACS, 6.0%). The likelihood of AKI 3 was highest in sepsis (6.9%) and alcoholic liver disease (ALD, 6.2%). The likelihood of any AKI was lowest in acute coronary syndrome (ACS, 6.0%) and COPD (7.7% As a proportion of AKI events, AKI 3 events were most likely in ALD (26.3% of AKI events, see Fig 1), and least likely in ACS (9.4%) and COPD (9.3%). Fig 1 also shows that as the risk of any AKI increased in medical diagnoses, the risk for AKI 3 also proportionately increased.

**Table 3:** Event counts for acute kidney injury by medical and surgical diagnosis.

Diagnosis	N	AKI 3			Any AKI	
		N	% AKI	% cases	N	% cases
Medical						
ACS	4266	24	9.4	0.6	255	6.0
ALD	581	36	23.5	6.2	153	26.3
CAP	7232	203	16.2	2.8	1253	17.3
COPD	4234	30	9.3	0.7	324	7.7
GIB	1615	25	16.4	1.5	152	9.4
HF	1652	57	16.5	3.5	346	20.9
Sepsis	1672	116	21.8	6.9	532	31.8
UTI	4800	147	18.2	3.1	808	16.8
Overall	26052	638	16.7	2.4	3823	14.7
Surgical						
Abscess	1352	1	5.2	0.1	19	1.4
Appendix	683	1	4.2	0.1	24	3.5
Chole	2261	24	9.9	1.1	242	10.1
ENT	657	2	22.2	0.3	9	1.4
NOF	1815	22	6.6	1.2	332	18.3
NTICB	2830	18	3.5	0.6	518	18.3
Panc	936	9	9.6	0.1	94	10.0
TICB	2026	8	2.8	0.4	282	13.9
Overall	12560	85	5.6	0.7	1520	12.1

**Table key:** HF–heart failure, ACS–acute coronary syndrome, CAP–community acquired pneumonia, COPD–chronic obstructive pulmonary disease, UTI–urinary tract infection, ALD–alcoholic liver disease, GIB–gastro-intestinal bleed, sepsis–sepsis (any source).

Chole—Cholecystitis/cholangitis, TICB—Traumatic Intra Cranial Bleed, NTICB- Non-Traumatic Intra Cranial Bleed, abscess–abscess (any source), Panc—acute pancreatitis, appendix—acute appendicitis, ENT (ear, nose and throat)–ENT (any source), NOF—Fractured (neck of femur) NOF. % AKI = percent of AKI events that were AKI 3, % cases = % of patients with selected diagnosis who had AKI 3 / any AKI.

There were 12,560 patient episodes within the 8 selected surgical diagnoses, with 1,520 (10.0%) AKI events. There were 85 AKI stage 3 events (0.6% of the overall population AKIs, 5.6% of the surgical diagnoses). Overall, surgical diagnoses were less likely to manifest AKI than medical diagnoses. Of the 8 diagnoses in which an AKI was most likely, only 2 were surgical diagnoses. For AKI 3, only 1 of the 8 diagnoses in which AKI 3 occurred most frequently was a surgical diagnosis.

**Fig 2:**
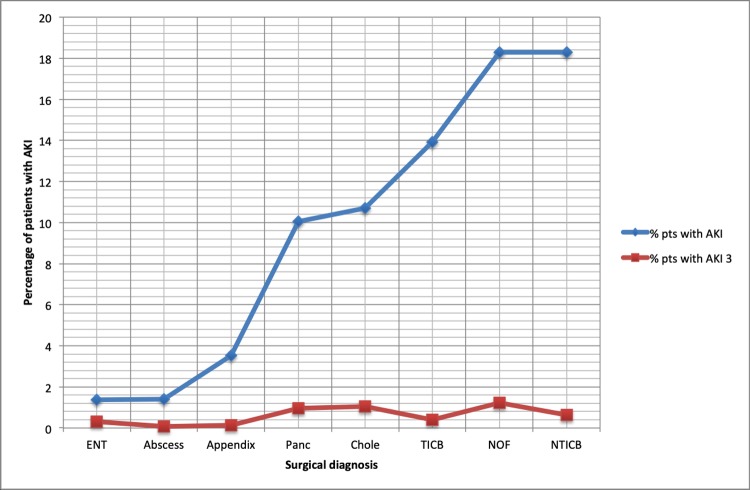
Percentage of patients with any acute kidney injury (AKI) or AKI 3 in each surgical diagnosis, ordered by increasing frequency of AKI. **Figure key**: Chole—Cholecystitis/cholangitis, TICB—Traumatic Intra Cranial Bleed, NTICB- Non-Traumatic Intra Cranial Bleed, abscess–abscess (any source), Panc—acute pancreatitis, appendix—acute appendicitis, ENT (ear, nose and throat)–ENT (any source), NOF—Fractured (neck of femur) NOF. % pts with AKI = percentage of patients with each diagnosis with AKI or AKI 3.

The likelihood of any AKI was highest in femoral neck fracture and NTICB (18.3% in both patient groups). The likelihood of AKI 3 was highest in femoral neck fracture (1.2%) and cholecystitis or cholangitis (1.1%). As a proportion of AKI events, patients with ENT diagnoses and pancreatitis were most likely to have AKI 3 (22.2% and 9.6% of admissions respectively). This data is, however, skewed by small numbers, as there were 2 AKI 3 events in 9 patients for ENT admissions, and 9 AKI 3 events for 94 pancreatitis admissions. The lowest incidence of any AKI occurred in patients with abscesses and ENT diagnoses. AKI 3 events were lowest in traumatic and non-traumatic intracranial bleeds (2.8% and 3.5% of admissions respectively). Unlike for medical diagnoses, as the risk of any AKI increased in surgical diagnoses, there was no corresponding increase in the likelihood of AKI 3. AKI 3 occurred in less than 2% of patients in all surgical diagnoses (Fig 2).

### AKI associated mortality

Overall mortality in the 8 chosen medical diagnoses was 5.1%, but there was a 5 fold increase to 27.7% for mortality with any AKI, and an 8 fold increase to 42.6% if an AKI 3 supervened. In comparison, the surgical incidence of mortality was lower, with a 1.9% overall mortality, 13.7% mortality in any AKI, and 29.4% mortality in AKI 3.

For medical diagnoses, the likelihood of death in any AKI was highest in community-acquired pneumonia (37.8%, compared with 8% for all community-acquired pneumonia), followed by sepsis (32.7%, compared with 14% for all sepsis patients). Mortality was lowest in any AKI in UTI (12.7%) and gastrointestinal bleed (17.8%). The likelihood of death in AKI 3 was highest in ALD (52.8%), followed by CAP (52.7%). The lowest risk of death in AKI 3 was also UTI (24.5%) and gastrointestinal bleed (28.0%).

For surgical diagnoses, the likelihood of death with and without AKI was generally lower than for medical diagnosis (any AKI 13.7% versus 27.7%, AKI 3 29.4% versus 42.6%). The likelihood of death in any AKI was highest in femoral neck fracture (18.4%), followed by traumatic intracranial bleed (TICB, 16.7%). Mortality was lowest in appendix and ENT surgery, where there were no deaths in either group. In diagnoses where deaths did occur, the lowest risk of death for any AKI was in patients with abscesses (5.3%). The likelihood of death in AKI 3 was highest in femoral neck fracture (54.5%), higher than in any medical diagnosis. The next highest likelihood of death in AKI 3 was in cholecystitis (29.2%). The lowest risk of death in diagnoses where deaths occurred was in TICB (12.5%) and NTICB (16.6%). A comparison of all mortality event rates across all medical and surgical diagnoses is found in Table 4, which includes mortality in the overall population for each diagnosis as a comparator.

**Table 4:** Event counts for death in medical and surgical admissions with AKI.

Diagnosis	Total	All patients		AKI 3		Any AKI	
		N	%	N	%	N	%
Medical							
ACS	4266	63	1.5	8	33.3	55	21.6
ALD	581	59	10.2	19	52.8	40	26.1
CAP	7232	581	8.0	107	52.7	474	37.8
COPD	4234	91	2.1	15	50.0	76	23.5
GIB	1615	34	2.1	7	28.0	27	17.8
HF	1652	137	8.3	27	47.4	110	31.8
Sepsis	1672	227	13.6	53	45.7	174	32.7
UTI	4800	139	2.9	36	24.5	103	12.7
Overall	26052	1331	5.1	272	42.6	1059	27.7
Surgical							
Abscess	1352	1	0.1	0	-	1	5.3
Appendix	683	0	-	0	-	0	-
Chole	2261	31	1.4	7	29.2	24	9.9
ENT	657	0	-	0	-	0	-
NOF	1815	73	4.0	12	54.5	61	18.4
NTICB	2830	71	2.5	3	16.6	68	13.1
Panc	936	9	1.0	2	22.2	7	7.4
TICB	2026	48	2.4	1	12.5	47	16.7
Overall	12560	233	1.9	25	29.4	208	13.7

**Table key:** HF–heart failure, ACS–acute coronary syndrome, CAP–community acquired pneumonia, COPD–chronic obstructive pulmonary disease, UTI–urinary tract infection, ALD–alcoholic liver disease, GIB–gastro-intestinal bleed, sepsis–sepsis (any source).

Chole—Cholecystitis/cholangitis, TICB—Traumatic Intra Cranial Bleed, NTICB- Non-Traumatic Intra Cranial Bleed, abscess–abscess (any source), Panc—acute pancreatitis, appendix—acute appendicitis, ENT (ear, nose and throat)–ENT (any source), NOF—Fractured (neck of femur) NOF. % AKI = percent of AKI events that were AKI 3, % cases = % of patients with selected diagnosis who had AKI 3 / any AKI.

Any AKI in medical admissions conveyed an adjusted OR of 4.7 (95% confidence intervals 4.3–5.2, p<0.001) for death compared to patients without AKI. AKI 3 had a OR of 6.2 (5.2–7.4, p<0.001) in comparison to patients with either AKI 1, AKI 2, or no AKI. In medical diagnoses, the highest increased risk for death in any AKI compared to no AKI was in acute coronary syndrome (ACS, 8.9 [5.8–13.5, p<0.001]), followed by heart failure (6.0 [4.3–8.4, p<0.001]). In AKI 3, the highest increased risk for death compared to all other patients was in ACS (12.8 [4.8–33.8, p<0.001]), and COPD (11.3 [5.1–24.4, p<0.001]). In medical diagnoses the lowest increase in risk for death in any AKI compared to no AKI was ALD (OR 3.0[1.7–5.2, p<0.001]) and sepsis (3.5 [2.6–4.6, p<0.001]). These were the medical diagnoses with the highest overall mortality (10% and 14% respectively) and the lower ORs here likely reflect the higher baseline mortality for these. For AKI 3, compared to all other patients, the lowest increase risk for death was again found in sepsis (4.2 [2.7–6.4, p<0.001]).

Any AKI in surgical diagnoses conveyed an adjusted OR of 1.8 (95% confidence intervals 1.5–2.1, p<0.001) for death in comparison to patients without AKI. In the selected surgical diagnoses, the highest risk for death in any AKI was in pancreatitis (OR 9.7 [2.5–37.4, p = 0.001]), which is significantly higher than the other surgical diagnoses and higher than any of the medical diagnoses. AKI 3 in the surgical diagnoses had a OR of 4.0 (2.4–6.5, p<0.001) for death compared to patients with AKI 1, AKI 2, or no AKI. This was highest in patients with femoral neck fracture (24.6 [8.9–67.9, p<0.001]) and pancreatitis (16.1 [2.2–119.6, p = 0.007]). These were both higher than the OR for death with AKI in any specific medical diagnosis. There were no deaths in patients with appendicitis or ENT diagnoses with any AKI or AKI 3, and there were no deaths in patients with abscesses and AKI 3. For patients with NTICB, both AKI and AKI 3 were associated with a lower risk of death than their respective comparator groups, although in the latter case this did not reach statistical significance. For any AKI, the OR was 0.6 (0.5–0.8, p = 0.003), and AKI 3 was 0.8 (0.2–2.9, p = 0.737). These results all detailed in Table 5.

**Table 5:** Comparative risk for death in medical and surgical admissions with AKI.

Diagnosis	Adjusted OR for death			
	AKI 3 versus all others		Any AKI versus no AKI	
	OR	95% CI	OR	95% CI
Medical				
ACS	12.8	4.8–3 3.8	8.9	5.8–13.5
ALD	7.1	3.3–14.8	3.0	1.7–5.2
CAP	5.7	4.2–7.7	3.9	3.4–4.6
COPD	11.3	5.1–24.4	6.6	4.7–9.2
GIB	5.9	2.1–16.6	4.2	2.3–7.6
HF	6.2	3.5–11.0	6.0	4.3–8.4
Sepsis	4.2	2.7–6.4	3.5	2.6–4.6
UTI	5.9	3.7–9.2	4.6	3.3–6.3
Overall	6.2	5.2–7.4	4.7	4.3–5.2
Surgical				
Abscess	-	-	1.1	0.1–20.5
Appendix	-	-	-	-
Chole	9.4	3.2–27.3	3.4	1.9–6.3
ENT	-	-	-	-
NOF	24.6	8.9–67.9	3.8	2.6–5.6
NTICB	0.8	0.2–2.9	0.6	0.5–0.8
Panc	16.1	2.2–119.6	9.7	2.5–37.4
TICB	1.1	0.1–9.0	1.7	1.2–2.5
Overall	4.0	2.4–6.5	1.8	1.5–2.1

**Table key**: HF–heart failure, ACS–acute coronary syndrome, CAP–community acquired pneumonia, COPD–chronic obstructive pulmonary disease, UTI–urinary tract infection, ALD–alcoholic liver disease, GIB–gastro-intestinal bleed, sepsis–sepsis (any source).

Chole—Cholecystitis/cholangitis, TICB—Traumatic Intra Cranial Bleed, NTICB- Non-Traumatic Intra Cranial Bleed, abscess–abscess (any source), Panc—acute pancreatitis, appendix—acute appendicitis, ENT (ear, nose and throat)–ENT (any source), NOF—Fractured (neck of femur) NOF. OR = odds ratio, 95% CI = 95% confidence interval

Where no result is given,—denotes unable to calculate due to lack of episodes

### AKI associated critical care admissions

There were 9001 admissions to critical care, with admission numbers greater in the surgical diagnoses (1852, 20.1%) than the medical diagnoses (606, 6.7%). Admission to critical care in patients with medical diagnoses and any AKI had a OR of 9.6 (95% confidence interval 8.6–10.8, p<0.001) in comparison to medical patients without AKI. For individual medical diagnoses, the highest OR for admission to critical care associated with AKI was for heart failure. Here, the adjusted OR was 37.2 (18.9–73.4, p<0.001). Overall for medical patients, those with an AKI 3 compared to any of AKI 1, AKI 2 or no AKI had a OR of 3.4 (2.7–4.1, p<0.001) for risk of admission to critical care. Patients with ACS had the highest OR at 8.6 (1.9–37.2, p = 0.004), followed by those with heart failure (7.8 [2.7–21.9, p<0.001]). Sepsis was associated with the lowest increased risk for critical care admission in both any AKI (6.8 [5.2–9.0, p<0.001]) compared to no AKI, and AKI 3 (2.2 [1.4–3.5, p = 0.001]) compared to other patients. This most likely reflects the higher baseline rate of adverse outcome in these diagnoses. The event rates for critical care admissions for each diagnosis, in the overall population, as well as those with any AKI and AKI 3, is found in Table 6.

**Table 6:** Comparative risk for critical care admission in medical and surgical admissions with AKI.

Diagnosis	Total	All patients		AKI 3		Any AKI N	
		N	%	N	%	N	%
Medical							
ACS	4266	46	1.1	2	4.3	21	45.7
ALD	581	35	6.0	9	25.7	21	60.0
CAP	7232	248	3.4	38	15.3	145	58.5
COPD	4234	27	0.6	3	11.1	15	55.6
GIB	1615	42	2.6	6	14.3	22	52.4
HF	1652	13	0.8	4	30.8	9	69.2
Sepsis	1672	123	7.4	30	24.4	87	70.7
UTI	4800	72	1.5	22	30.6	52	72.2
Overall	26052	606	2.3	114	18.8	372	61.4
Surgical							
Abscess	1352	8	0.6	2	25.0	0	0.0
Appendix	683	40	6.1	0	0.0	10	25.0
Chole	2261	117	5.2	8	6.8	51	43.6
ENT	657	3	0.5	0	0.0	0	0.0
NOF	1815	110	6.1	3	2.7	43	39.1
NTICB	2830	975	34.5	12	1.2	340	34.9
Panc	936	54	5.8	5	9.3	25	46.3
TICB	2026	545	26.9	5	0.9	184	33.8
Overall	12560	1852	14.7	35	1.9	653	35.3

**Table key**: HF–heart failure, ACS–acute coronary syndrome, CAP–community acquired pneumonia, COPD–chronic obstructive pulmonary disease, UTI–urinary tract infection, ALD–alcoholic liver disease, GIB–gastro-intestinal bleed, sepsis–sepsis (any source).

Chole—Cholecystitis/cholangitis, TICB—Traumatic Intra Cranial Bleed, NTICB- Non-Traumatic Intra Cranial Bleed, abscess–abscess (any source), Panc—acute pancreatitis, appendix—acute appendicitis, ENT (ear, nose and throat)–ENT (any source), NOF—Fractured (neck of femur) NOF. % AKI = percent of AKI events that were AKI 3, % cases = % of patients with selected diagnosis who had AKI 3 / any AKI.

The OR for admission to critical care in any surgical diagnosis for patients with any AKI was 4.3 (3.8–4.9, p<0.001) compared to patients without AKI. The surgical diagnosis with the highest OR was appendicitis (6.0, [2.1–17.7, p = 0.001]), followed by pancreatitis (5.4 [2.8–10.6, p<0.001]). The lowest increased risk among surgical diagnoses was in NTICB (2.4 [1.9–3.0, p<0.001]) and femoral neck fracture (2.5 [1.6–3.8, p<0.001]). Patients with a surgical diagnosis and an AKI 3 had a OR of 2.1 (1.3–3.4, p = 0.002) for risk of admission to critical care compared to surgical patients with any of AKI 1, AKI 2, or no AKI. Of specific surgical diagnoses, pancreatitis had the highest increased risk in the presence of AKI 3 at 8.1 (1.8–35.3, p = 0.006). The lowest OR for admission to critical care in the surgical diagnoses with AKI 3 was in patients with a femoral neck fracture (1.3 [0.4–5.0, p = 0.675]). These comparisons are shown in Table 7.

**Table 7:** Comparative risk for critical care admission in medical and surgical admissions with AKI.

Diagnosis	Adjusted OR for death						
	AKI 3 versus all others				Any AKI versus no AKI		
	OR	95% CI	p		OR	95% CI	p
Medical							
ACS	8.6	1.9–37.2		0.004	9.4	6.0–14.9	<0.001
ALD	2.7	1.2–5.9		0.018	12.7	7.3–22.1	<0.001
CAP	3.3	2.2–4.6		<0.001	8.3	6.9–10.0	<0.001
COPD	4.5	1.3–14.8		0.015	13.5	7.9–22.8	<0.001
GIB	3.1	1.2–7.8		0.02	8.2	5.1–13.2	<0.001
HF	7.8	2.7–21.9		<0.001	37.2	18.9–73.4	<0.001
Sepsis	2.2	1.4–3.6		<0.001	6.8	5.2–9.0	<0.001
UTI	3.8	2.4–5.9		<0.001	17.1	12.8–23.0	<0.001
Overall	3.4	2.7–4.1		<0.001	9.6	8.6–10.8	<0.001
Surgical							
Abscess	-	-		-	4.1	0.6–29.0	0.163
Appendix	-	-		-	6.0	2.1–17.7	0.001
Chole	4.5	1.6–11.6		0.002	3.9	2.6–6.1	<0.001
ENT	-	-		-	-	-	-
NOF	1.3	0.4–5.0		0.675	2.5	1.6–3.8	<0.001
NTICB	1.6	0.5–4.9		0.416	2.4	1.9–3.0	<0.001
Panc	8.1	1.8–35.3		0.006	5.4	2.8–10.6	<0.001
TICB	1.6	0.3–8.1		0.529	4.5	3.3–6.1	<0.001
Overall	2.1	1.3–3.4		0.002	4.3	3.8–4.9	<0.001

**Table key**: HF–heart failure, ACS–acute coronary syndrome, CAP–community acquired pneumonia, COPD–chronic obstructive pulmonary disease, UTI–urinary tract infection, ALD–alcoholic liver disease, GIB–gastro-intestinal bleed, sepsis–sepsis (any source).

Chole—Cholecystitis/cholangitis, TICB—Traumatic Intra Cranial Bleed, NTICB- Non-Traumatic Intra Cranial Bleed, abscess–abscess (any source), Panc—acute pancreatitis, appendix—acute appendicitis, ENT (ear, nose and throat)–ENT (any source), NOF—Fractured (neck of femur) NOF. OR = odds ratio, 95% CI = 95% confidence interval, p = p value

## Discussion

The presented data provide granularity to, and add to our current understanding of, the epidemiology and associated risks of AKI in specific medical and surgical inpatient populations.

### AKI incidence

The incidence of any AKI and AKI 3 was highest in sepsis, reflecting the known complication of sepsis and haemodynamic compromise. In sepsis management it needs to be emphasized that almost one third of patients will suffer an AKI, and that fluid optimization, source control, a careful approach to use of nephrotoxic medication, and regular ongoing review are important. [14,15]

Overall, there were smaller numbers of patients suffering any stage of AKI in the surgical population. The majority were confined to 2 diagnoses: femoral neck fracture and non-traumatic intracranial bleed. Very few surgical patients were found to suffer AKI 3, which is likely due to a combination of confounding issues. The ICD-10 coded diagnoses are discharge diagnoses and patients may be too unwell to be investigated for, or to have their management changed by, a surgical diagnosis. This may not therefore be their primary coded discharge diagnosis. In most NHS hospital models, surgical patients are pre-selected by the surgical team and are composed of more medically fit patients who are suitable for anaesthetic.[16] This consequently leaves those with greater age and comorbid burden who would be destined for conservative management of a potentially surgical problem under the care of the medical teams.

An improved understanding of the incidence and risk of AKI and related outcomes in relation to the specific underlying condition could support the design of bespoke teaching packages and facilitate targeting of resources. It could direct education to areas or teams that deal with higher numbers of AKI and AKI 3 to maximize effectiveness of AKI prevention and overall patient care. The scarcity of AKI events in patients with abscesses, appendicitis, pancreatitis or ENT diagnoses also argues in support of early and regular medical or renal input, as expertise is unlikely to be maintained in those fields. This supports an argument not just for personalised care for patients but also personalised education for their doctors.

### AKI associated mortality

The high likelihood of death in community-acquired pneumonia and alcoholic liver disease reflects the overall physiological condition of patients with these diagnoses. These two diagnoses are suggestive of progressive single organ failure and are associated with significant mortality with or without sepsis, and with or without AKI.[17]

Most significantly for the surgical specialties and the orthogeriatrics team, not only were patients with a femoral neck fracture the most likely to get an AKI, but their likelihood of death was also highest, with the lowest chance for critical care admission. Again, this likely reflects the underlying frailty of the patient, irrespective of chronological age and the severity of the intercurrent illness causing AKI. However, it highlights important discrepancies in management of different surgical patients and deserves further research to understand any inequalities in care (e.g. access of aged patients to higher level care).

The majority of admissions for patients with coded diagnoses of ACS and COPD are short stays with mild exacerbations of disease. Therefore, if these patients have a significant additional pathology or haemodynamic disturbance that leads to an intercurrent AKI, then this would plausibly be linked to a higher risk of death.

### AKI associated critical care admissions

Admissions to critical care are positively skewed towards surgical patients. Surgical patients have a low threshold for pre-emptive critical care admission compared to medical diagnoses. The Royal College of Surgeons criteria states that if patients have a >10% chance of mortality post-operatively they should be cared for in a critical care facility.[18] This may also contribute to the reduced OR for death seen in surgical patients who suffer AKI in comparison to medical patients suffering an AKI. This could lend support to recommend therefore that medical patients with the highest risk of death should also be transferred early to critical care.

The level of granularity provided here could be used to model an inpatient journey or ‘forecast’ predictable care needs. It provides weight and data regarding level of care likely to be required for different specialty components of the hospital population, geographical location for this need, and specialty team input required. Feasibly, this could be used as a trigger threshold for acute medical review or renal input into surgical specialties in a timely manner, or as a regular occurrence built into job plans. It could aid planning during winter pressures for elective procedures depending on likely critical care bed availability or projected occupancy. The data could also lend support for provision of palliation or bereavement provision and advice both in and out of hours.

### Limitations

Salford Royal NHS Foundation Trust has a long history of digital excellence and has won awards for digital maturity. However, the ICD 10 codes and coding practice are prone to incompleteness or redundancy when linked to primary care. This may affect the categories of diagnosis and count of comorbidities.

There is an inherent selection bias for patients undergoing surgery or selected to be under surgical care. If a patient with multiple co-morbidities were to be considered for conservative management of a surgical pathology, the medical team may look after the patient.

Critical care covers a 24-bedded unit for general patients, a neurosurgical unit (8 beds) and a surgical high-dependency unit (8 beds). In addition to its critical care services, the Trust has a medical high care unit that has 8 beds and provides high-flow oxygen, non-invasive ventilation and cardiac monitoring. This model often supports patients unsuitable for escalation to critical care but who still are categorised as critical care in terms of coding. Therefore, the data for medical admissions to critical care may be slightly overestimated in comparison to similar tertiary teaching hospitals that lack similar units.

### Conclusion

Increasingly, AKI should be considered ‘an illness barometer’ that affects the outcome of any underlying condition. The kidneys can be seen as sentinel organs: their dysfunction is a marker of the unwell patient. In conjunction with clinical acumen, an AKI alert in combination with an early warning score could be an indication for early daily senior medical input. The quantitative data provided here can also support managerial decisions in terms of bed management—for example, with an AKI defining patients who should not be outlied (placed outside an acute medical ward).

The clinical impact of AKI differs across medical and surgical diagnoses, but is a significant contributor to the risk for death and critical care admission. This body of work may indicate a benefit to a more diagnosis-specific stratified approach to personalised AKI care after acute admission in respect of decisions for investigations and management, escalation of care, prompt referral or palliation.
